# Evaluation of the efficacy of physical agent modalities in patients with fractures: a systematic review and network meta-analysis

**DOI:** 10.3389/fmed.2025.1646903

**Published:** 2025-10-29

**Authors:** Lei Li, Lishi Yang, Yue Yang, Jiayi Zhu, Rongnan Shi, Qi Deng, Jianxiong Wang, Fuhua Sun

**Affiliations:** ^1^Rehabilitation Medicine Department, The Affiliated Hospital of Southwest Medical University, Luzhou, Sichuan, China; ^2^Department of Oncology, The Affiliated Hospital of Southwest Medical University, Luzhou, Sichuan, China; ^3^Department of Rehabilitation Medicine, Southwest Medical University, Luzhou, Sichuan, China; ^4^Rehabilitation Medicine and Engineering Key Laboratory of Luzhou, Luzhou, Sichuan, China

**Keywords:** physical agent modalities, fracture, bone healing, pain relief, network meta-analysis

## Abstract

**Background:**

Fractures are increasing due to ageing populations. Physical agent modalities, a non-invasive treatment, enhances healing and reduces non-union risk.

**Objective:**

This meta-analysis evaluates the effectiveness of physical agent modalities in patients with fractures and compares the outcomes of different interventions on healing and pain relief.

**Methods:**

Articles published up to April 2025 were retrieved from PubMed, Embase, and Web of Science. Two authors independently reviewed and extracted data from randomized controlled trials assessing seven types of physical agent modalities: Electrical Stimulation (ES), Pulsed Electromagnetic Fields Stimulation (PEMFS), Ultrasound Therapy (UST), Low-Level Laser Therapy (LLLT), Magnetic Stimulation (MS), Extracorporeal Shock Wave Therapy (ESWT), and Capacitively Coupled Electric Field Stimulation (CCEFS). Standard meta-analysis and network meta-analysis (NMA) were performed for three outcomes: Pain Relief Difference, Time to Complete Fracture Healing (days), and Number of Cases Achieving Complete Fracture Healing. Cumulative ranking curves (SUCRA) scores were calculated for each therapy, with data presented as mean differences (MD) and 95% confidence intervals (CI).

**Results:**

This meta-analysis includes 39 studies with 2,379 participants. The standard meta-analysis results show that physical agent modalities can markedly enhance fracture healing, with significant pain relief (MD = 1.30, 95% CI: 0.61, 1.99), *P* = 0.0002, shorter time to complete fracture healing (days) (MD = −21.58, 95% CI: −31.05, −12.11), *P* < 0.0001, and more number of cases achieving complete fracture healing (RR = 1.37, 95% CI: 1.17, 1.60), *P* < 0.0001. However, the NMA findings indicate that most direct or indirect comparisons between different physical agent therapies yield pooled effect sizes whose 95% confidence intervals include the null value (0 or 1), showing no significant differences between groups. SUCRA rankings revealed that LLLT (87.5%) and ES (80.8%) were more effective in pain relief, while UST (82.9%) and CCEFS (99.9%) excelled in promoting fracture healing.

**Conclusion:**

LLLT, ES, UST, and CCEFS may yield improved outcomes for fracture patients; however, further high-quality, large-scale randomized controlled trials are required to validate these findings.

## 1 Introduction

Bone fractures, a prevalent condition, are currently witnessing an upward trend in global incidence (1), predominantly linked to population ageing and increased life expectancy. According to United Nations research, the global population aged ≥65 years is projected to reach 1.5 billion by 2050 (2, 3). As the ageing process accelerates, the incidence of fractures continues to rise, imposing substantial socioeconomic burdens on individuals, families, and societies (4). Fracture patients often experience major symptoms such as restricted mobility and acute pain (5), which typically ease over a two-month period, with most individuals reaching optimal recovery within 3–6 months (6). Depending on the severity of the fracture, treatment may involve either conservative management or surgery. For non-displaced fractures, conservative treatment generally leads to good outcomes; according to the BMJ Clinical Practice Guidelines^1^, recovery usually takes 3–4 weeks with relatively rapid pain relief. Displaced fractures, on the other hand, often require surgical intervention, which can support earlier functional recovery (around 10–14 weeks) (7), but may also carry about a 20% risk of non-union and persistent pain, potentially affecting quality of life (8–11). It is important to note that the duration of pain relief following surgery varies considerably among individuals (12).

Bone healing following a fracture is a complex physiological process that is typically divided into four stages: the fracture and inflammatory phase, the angio-mesenchymal phase, the bone formation phase, and the bone remodeling phase. Although each phase possesses distinct characteristics, they often occur alternately and exhibit a degree of overlap (13). The process of bone healing is influenced by a variety of factors, including the nature and extent of the injury, the damage to the surrounding soft tissues, blood supply, the differentiation capacity of osteoblasts, and the cellular microenvironment as internal factors. Additionally, external factors such as the stability of fracture fixation, the gap between fracture ends, the inflammatory response, and external physical stimuli also play a significant role in the healing process (14). In the final stage of bone healing, known as the bone remodeling phase, approximately 5–10% of long bone fractures may experience non-union (15). In cases of delayed healing or non-union during the fracture healing process, surgical intervention is often required. Autologous bone grafting, regarded as the gold standard for the treatment of fractures and bone defects, has been widely employed in clinical practice (16). In addition to surgical treatment, non-invasive physical agent modalities such as electrical stimulation, electromagnetic stimulation, low-intensity pulsed ultrasound, and low-level laser therapy have been shown to facilitate the acceleration of fracture healing and have gained widespread recognition in clinical practice. These adjunctive therapies provide effective supplementary strategies for optimizing the healing of fractures (17–20).

Most randomized controlled trials (RCTs) use standard care as a control, while few directly compare distinct physical agent modalities modalities. Traditional meta-analyses typically allow for the comparison of only two treatment methods at a time, failing to provide comprehensive evidence regarding the relative efficacy of various interventions for fracture healing. Network meta-analysis (NMA) addresses this limitation by facilitating simultaneous comparisons of multiple treatment options and enabling the ranking of each intervention based on various outcomes. This approach offers clinicians a clear, evidence-based framework for treatment decisions, thereby assisting in making informed and scientifically sound clinical choices when addressing complex cases of delayed healing or non-union (21).

Consequently, we conducted a NMA aimed at synthesizing the existing evidence to compare the efficacy of different physical agent modalities in promoting fracture healing and alleviating pain. The specific objective is to identify the most effective physical agent modalities approach, thereby providing robust support for clinical decision-making and assisting in the optimization of treatment strategies for fracture healing.

## 2 Methods

The protocol was registered with PROSPERO under registration number CRD420251030229.

### 2.1 Search strategy

This NMA adheres to the Preferred Reporting Items for Systematic Reviews and Meta-Analyses (PRISMA) guidelines (22). As of April 2025, we conducted a comprehensive search for relevant literature in the PubMed, Embase, and Web of Science databases. Search strategies were developed for each of the three databases (See Supplementary Table 1 for details). Two authors (LL and SFH) independently conducted literature searches and screenings, with any discrepancies resolved through mutual discussion. To augment potential relevant studies, the authors also examined the references of the included literature. The language was restricted to English, with no date limitations applied.

### 2.2 Exclusion and inclusion criteria

Studies that met the following criteria were included: (1) Patients with fractures or delayed healing following a fracture, regardless of fracture location and severity. (2) Physical agent modalities involving one or more of the following: Electrical Stimulation (ES), Pulsed Electromagnetic Fields Stimulation (PEMFS), Ultrasound Therapy (UST), Low-Level Laser Therapy (LLLT), Magnetic Stimulation (MS), Extracorporeal Shock Wave Therapy (ESWT), and Capacitively Coupled Electric Field Stimulation (CCEFS). (3) Control groups receiving either placebo stimulation or standard treatment alone. (4) studies reporting at least one outcome of interest, including pain, time to complete fracture healing, and the number of cases of complete fracture healing. (5) Randomized controlled trial (RCT) design. We excluded: ➀ Non-human studies; ➁ Studies lacking quantifiable outcome measures; ➂ Studies not involving disease models; ➃ Case reports, reviews, editorials, commentaries, conference abstracts, and articles not in English.

### 2.3 Data extraction

Two authors independently conducted eligibility assessments on the retrieved articles, initially excluding irrelevant studies based on their titles and abstracts. The remaining articles were then downloaded for a comprehensive review of the full texts, from which data were extracted for the eligible studies, including the first author’s name, publication year, country/region, participant characteristics (sample size, mean age, fracture location), interventions, follow-up duration, and outcomes of interest (Table 1). When extracted data were presented as medians and interquartile ranges, we applied Hozo’s formula to convert them into means and standard deviations (23). In cases of discrepancies, discussions were held to reach consensus. If any required information was missing, the corresponding author of the article was contacted via email.

**TABLE 1:** Main characteristics of included studies.

References	Country/ Regions	Mean age (years) (SD) Intervention/Comparator		Intervention/ Comparator	Sample size (n) (intervention/comparator) Intervention/Comparator		Fracture site	Follow-up	Outcomes
Acosta-Olivo et al. (36)	Mexico	54.8 (13.07)		Laser acupuncture/acupuncture	13	13	Wrist Bone	1 month	Pain relief difference
Barker et al. (37)	UK	>18	>18	Magnetic field/placebo stimulation	9	7	Tibia	12 month	Number of cases achieving complete fracture healing
Beck et al. (38)	Australia	28.33 (7.68)	26.09 (7.99)	Capacitively coupled electric field/placebo stimulation	22	21	Tibia	2 month	Time to complete fracture healing
Busse et al. (39)	Canada	37.1 (13.2)	39.1 (14.6)	LIPUS/placebo stimulation	214	201	Tibia	1 year +	Number of cases achieving complete fracture healing
Chang et al. (40)	Taiwan	33.64 (7.82)	30.56 (9.61)	Laser/placebo stimulation	25	25	Wrist and Hand	2 week	Pain relief difference
Cheing et al. (41)	China	63.8 (12.6)	60.3 (20.2)	Electromagnetic field/placebo stimulation	23	22	Distal radius	5 day	Pain relief difference
Duran et al. (42)	Istanbul	58.9 (10.7)	62.0 (9.5)	IFC/placebo stimulation	18	17	Proximal humeral	4 month +	Pain relief difference
Elboim-Gabyzon et al. (43)	Israel	80.26 (9.83)	78.06 (8.45)	TENS/placebo stimulation	23	18	Hip	5 day	Pain relief difference
Elsebahy et al. (44)	Egypt	5 ∼ 8		LIPUS/none	15	15	supracondylar	1 month +	Time to complete fracture healing
Factor et al. (45)	Israel	58 (13.25)	59 (16.75)	Electromagnetic field/placebo stimulation	14	13	Distal radius	3 month	Number of cases achieving complete fracture healing; time to complete fracture healing
Factor et al. (46)	Israel	49	59	Electromagnetic field/placebo stimulation	11	14	Distal radius	6 month	Number of cases achieving complete fracture healing
Fourie et al. (47)	South Africa	35 (11)	31 (13.25)	IFC/placebo stimulation	41	35	Tibial shaft	2 years +	Time to complete fracture healing
Gopalan et al. (48)	India	28 (7.291)	26.75 (8.723)	LIPUS/none	20	20	Mandibular	3 month	Number of cases achieving complete fracture healing
Gorodetskyi et al. (49)	Russia	71.5 (2)	70.8 (3)	Non-invasive interactive neurostimulation/placebo stimulation	30	30	Trochanteric of the femur	10 day	Pain relief differences
Gorodetskyi et al. (50)	Russia	35.3 (9)	38.4 (9)	Non-invasive interactive neurostimulation/placebo stimulation	30	30	Ankle	11 day	Pain relief difference
Hannemann et al. (51)	Netherlands	35 (13)	34 (14.75)	Electromagnetic field/placebo stimulation	51	51	Scaphoid	1 year +	Time to complete fracture healing
Hannemann et al. (52)	Netherlands	44.3 (17)	37.7 (13.25)	Electromagnetic field/placebo stimulation	24	29	Scaphoid	1 year +	Time to complete fracture healing
Heckman et al. (53)	USA	36 (2.3)	31 (1.8)	LIPUS/placebo stimulation	33	34	Tibia	9 month +	Number of cases achieving complete fracture healing; time to complete fracture healing
Kristiansen et al. (54)	USA	54 (3)	28 (2)	LIPUS/placebo stimulation	30	31	Distal radial	3 month +	Number of cases achieving complete fracture healing; time to complete fracture healing
Liu et al. (55)	China	61.5 (2.1)	63.5 (1.2)	Electromagnetic field/placebo stimulation	40	42	Vertebral	3 month +	Pain relief difference
Liu et al. (56)	China	67.9 (5.58)	65.7 (6.09)	LIPUS/none	41	40	Distal radius	1 month +	Time to complete fracture healing
Martinez-Rondanelli et al. (57)	Colombia	31 (10)	29 (9)	Electromagnetic field/placebo stimulation	32	31	Diaphyseal femoral	6 month	Number of cases achieving complete fracture healing
Mohajerani et al. (58)	Iran	37.06 (10.6)	37 (10.7)	Electromagnetic field/none	16	16	Mandibular	2 week	Pain relief difference
Moncada et al. (59)	Colombia	30.2		Magnetic field/placebo stimulation	32	32	Femoral shaft	6 month	Number of cases achieving complete fracture healing
Oncel et al. (60)	Turke	44 (15)	40 (16)	TENS/placebo stimulation	25	25	rib	3 day	Pain relief difference
Patel et al. (61)	India	15 ∼ 35		LIPUS/none	14	14	Mandibular	1 month +	Pain relief difference
Piazzolla et al. (62)	Italy	73.6 (7.82)	72.88 (6.09)	Capacitively coupled electric field/none	33	33	Vertebral	6 month	Pain relief difference
Ricardo et al. (63)	Cuba	26.7		LIPUS/placebo stimulation	10	11	Scaphoid	2.3 years	Time to complete fracture healing
Santana-Rodríguez et al. (64)	Saudi Arabia	64 (13.1)	58.9 (17.3)	PUS/placebo stimulation	24	23	Rib	6 month	Pain relief difference
Schofer et al. (65)	Germany	42.6 (14.6)	45.1 (11.9)	LIPUS/placebo stimulation	51	50	Tibia	4 month	Number of cases achieving complete fracture healing
Scott et al. (66)	UK	40 (9.05)	46 (20.09)	Capacitively coupled electric field/placebo stimulation	10	11	Long bones	9 month	Number of cases achieving complete fracture healing
Sharrard et al. (67)	UK	34.7 (17.66)	45.4 (14.76)	Electromagnetic field/placebo stimulation	20	25	Tibia	3 month	Number of cases achieving complete fracture healing
Shi et al. (68)	China	41.1 (14.5)	38.4 (11.6)	Electromagnetic field/placebo stimulation	31	27	Long bones	4 month +	Number of cases achieving complete fracture healing
Simonis et al. (69)	UK	31.7 (14.6)	32.3 (16.3)	Electrical stimulation/placebo stimulation	18	16	Tibia	6 month	Number of cases achieving complete fracture healing
Streit et al. (70)	USA	47 (9.75)		Electrical stimulation/placebo stimulation	5	3	Metatarsal	5 month +	Time to complete fracture healing
Wang et al. (71)	Taiwan	35.5 (16.0)	35.4 (19.2)	Shock wave/none	27	30	Long bones	12 month	Pain relief difference; number of cases achieving complete fracture healing
White et al. (72)	Canada	27.1 (9.4)	26.5 (12.1)	LIPUS/placebo stimulation	69	73	Scaphoid	2.4 years	Number of cases achieving complete fracture healing
Wu et al. (73)	China	43.1 (9.6)	42.5 (8.2)	ST + PNF + TEAS/ST + PNF	20	20	Tibial plateau	1 month +	Pain relief difference
Yadav et al. (74)	India	Unclear	Unclear	Ultrasound/placebo stimulation	39	28	Tibia	1 month +	Number of cases achieving complete fracture healing

### 2.4 Risk of bias

The risk of bias in the included studies was assessed using the Cochrane Risk of Bias Tool (RoB 2.0)^2^ (24) across six domains: the randomization process, deviations from intended interventions, missing outcome data, measurement of the outcome, selection of the reported results, and other sources of bias. The two authors independently rated each study as “low risk,” “high risk,” or “some concerns” for each of the aforementioned domains. Any discrepancies that arose during the review process were resolved through discussion or negotiation between the two authors.

### 2.5 Statistical analysis

In this study, for dichotomous outcomes, we reported risk ratios (RR) with 95% credible intervals, while for continuous outcome variables, we reported mean differences (MD) with 95% confidence intervals (CIs). Traditional meta-subgroup analyses were conducted using Review Manager 5.4.1, while calculations and visualizations were carried out using R 4.4.3 (R Foundation for Statistical Computing) and Stata SE 15.1 (StataCorp, College Station, TX). Given the heterogeneity between trials, we employed a Bayesian hierarchical random effects model for multiple comparisons (25, 26). Based on the theory of the likelihood function and certain initial assumptions, we performed Markov Chain Monte Carlo (MCMC) simulations using R 4.4.3, with 500,000 iterations and 20,000 for annealing to investigate posterior distributions (27–29). We assessed model goodness-of-fit by calculating the deviance information criterion (DIC) and employed the node splitting method to compare the consistency of direct and indirect evidence for each comparison (30). To address heterogeneity in the study, a random effects model was employed, and the degree of heterogeneity was quantified using the I^2^ statistic. To rank the interventions, we calculated the Surface Under the Cumulative Ranking Curve (SUCRA) probability values, which range from 0 to 1, with higher values indicating that the intervention is more likely to be the most effective (31, 32). A network diagram was created to analyse the geometrical structure of the intervention network and to identify potential biases, with the size of the nodes representing the number of participants in each group and the thickness of the lines reflecting the number of studies. A conjugate prior distribution was used for the Bayesian network meta-analysis (NMA), and a ranking table was generated to illustrate the comparisons of each pair of interventions for each outcome. Pairwise meta-analyses were conducted using the DerSimonian-Laird random effects model to estimate the variance of heterogeneity and obtain direct evidence (33). Finally, we utilized a comparison-adjusted funnel plot to assess potential publication bias (34, 35). Furthermore, sensitivity analyses were performed to explore their potential impact on the conclusions.

## 3 Results

### 3.1 Description of the included studies

**FIGURE 1:**
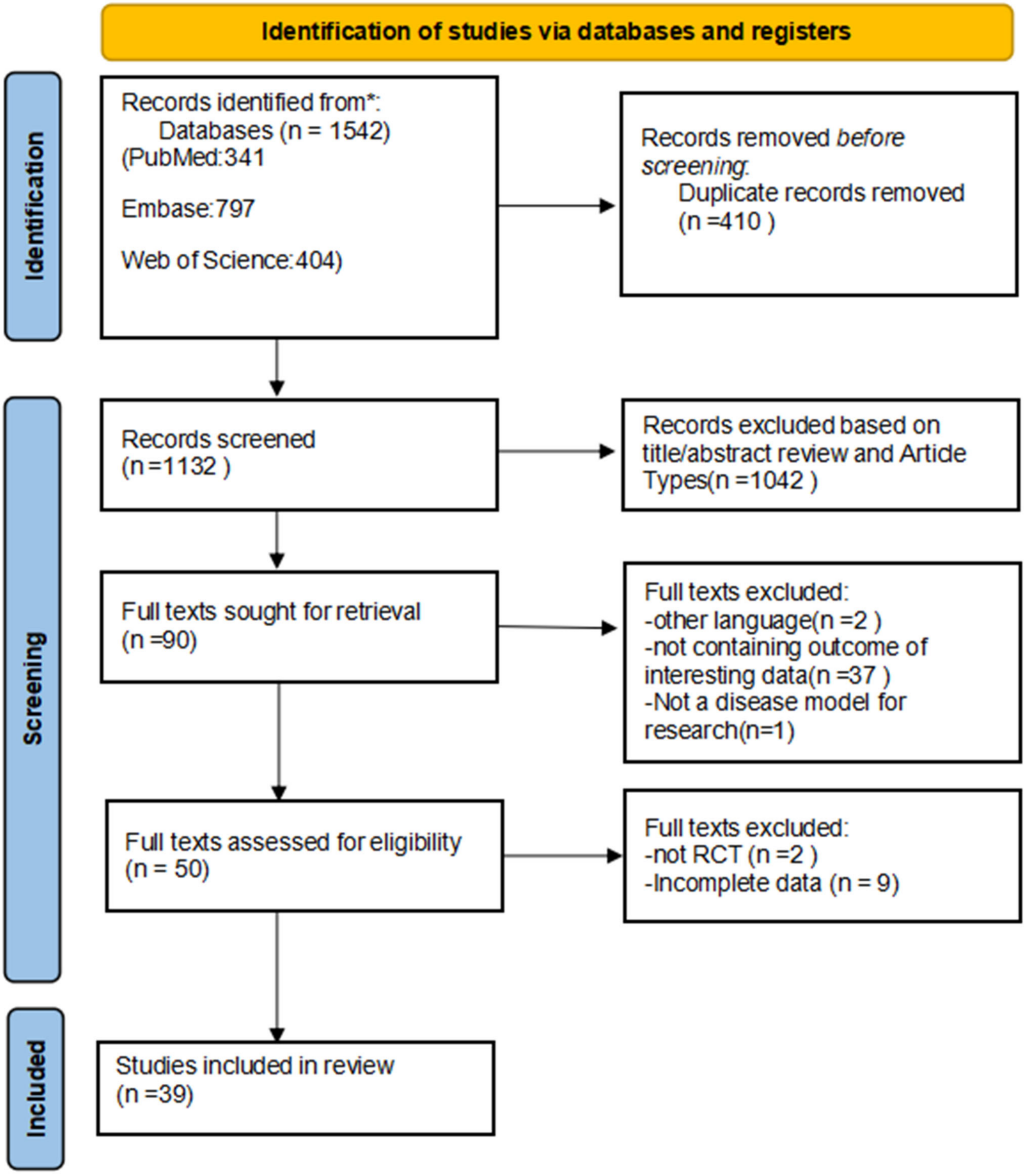
Flow diagram of the preferred reporting items for systematic reviews and meta-analyses (PRISMA) criteria. RCTs, randomized controlled trials.

We searched the PubMed, Embase, and Web of Science databases, identifying 341, 797, and 404 articles, respectively. After removing 410 duplicate articles, a total of 1,132 articles were identified. Based on title and abstract screening, 1,042 articles were excluded, leaving 90 articles that underwent full-text review, of which 39 articles met the eligibility criteria for our systematic review and NMA (36–74). The detailed PRISMA flow chart is presented in Figure 1.

Table 1 summarizes the characteristics of the 39 studies. Among these, 15 studies assessed pain improvement before and after physical agent modalities, 13 studies reported the time to complete fracture healing, and 17 studies documented the number of patients with fully healed fractures. Three studies evaluated capacitively coupled electric field stimulation, nine studies assessed electrical stimulation (ES), two studies investigated magnetic stimulation (MS), ten studies examined pulsed electromagnetic field stimulation (PEMFS), two studies focused on low-level laser therapy (LLLT), one study assessed extracorporeal shockwave therapy (ESWT), and twelve studies investigated ultrasound therapy (UST). This research was conducted across multiple countries, including China, the United States, Russia, Israel, Colombia, and India, and included 2,379 participants, with ages ranging from 5 to 72 years. The fracture sites were diverse, comprising 2.90% for humeral fractures (reported in two articles), 8.34% for femoral fractures (reported in three articles), 2.67% for ankle fractures (reported in one article), 14.18% for scapular fractures (reported in four articles), 10.66% for radial fractures (reported in five articles), 40.30% for tibial fractures (reported in ten articles), 1.82% for hip fractures (reported in one article), 4.32% for rib fractures (reported in two articles), 2.23% for carpal fractures (reported in two articles), 4.46% for mandibular fractures (reported in three articles), 0.4% for metatarsal fractures (reported in one article), and 6.60% for vertebral fractures (reported in two articles). Additionally, three studies reported on long bone fractures involving a total of 136 participants. Finally, the follow-up durations for the three outcome measures varied: the pain relief difference ranged from 5 days to 6 months; the time to complete fracture healing ranged from 1 month to 2.3 years; and the number of cases achieving complete fracture healing ranged from 1 month to 2.4 years.

### 3.2 Risk of bias

**FIGURE 2:**
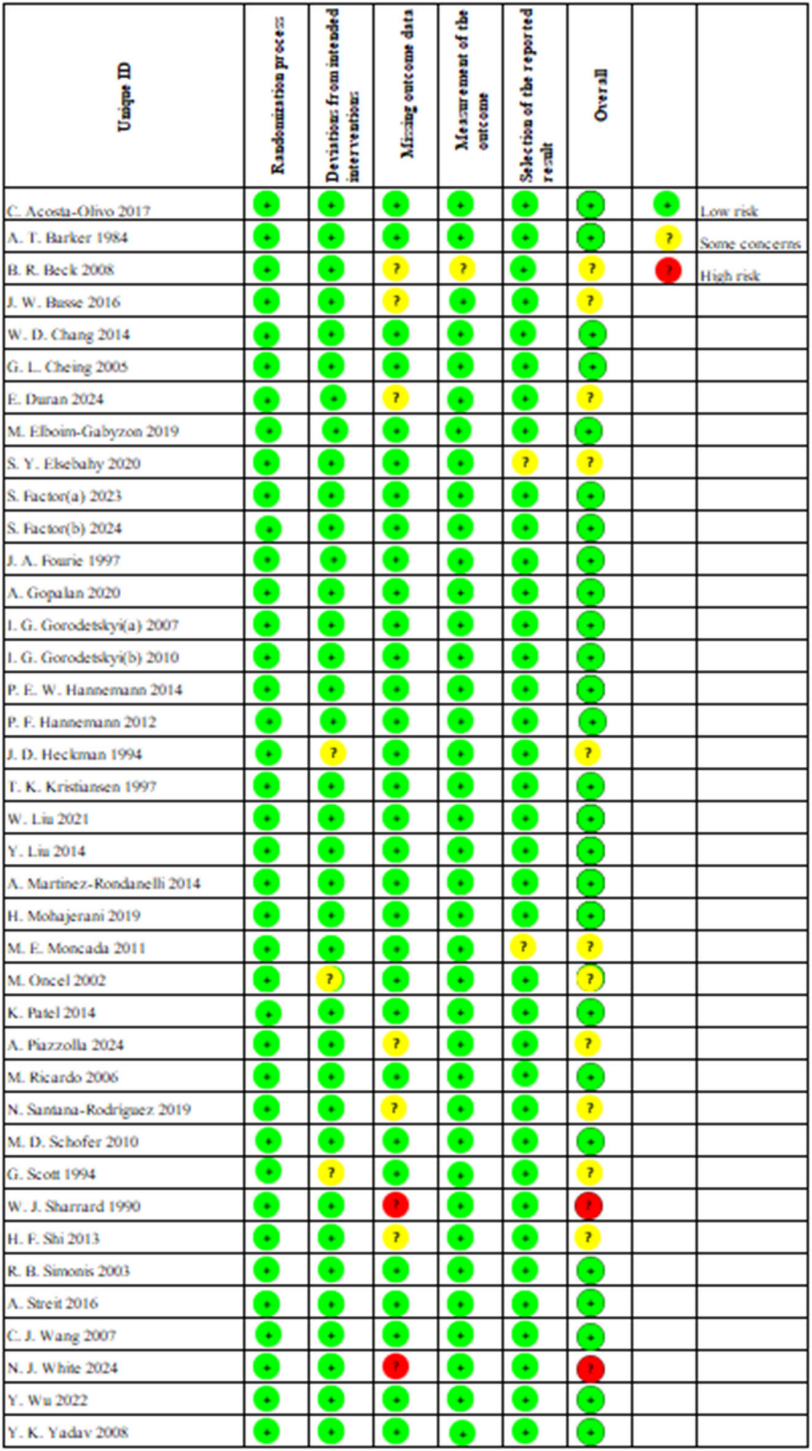
Traffic light plot for the risk-of-bias assessment of included trials.

We conducted an assessment of the risk of bias, the results of which are illustrated in Figure 2. Among the studies, 26 indicated a low risk of bias, 11 reported a moderate risk, and 2 demonstrated a high risk. All studies reported randomization. However, J. D. Heckman et al. (53), M. Oncel et al. (60) and G. Scott et al. (66) conducted unplanned treatments during the intervention phase, leading to their classification as moderate risk. W. J. Sharrard et al. (67) and N. J. White et al. (72) were deemed high risk due to individual participants withdrawing from the trial as a result of the intervention. Furthermore, B. R. Beck et al. (38), J. W. Busse et al. (39), E. Duran et al. (42), A. Piazzolla et al. (62), N. Santana-Rodríguez et al. (64) and H. F. Shi et al. (68) were classified as moderate risk due to dropout or loss to follow-up for personal reasons, whereas all other studies reported no loss of outcome data. B. R. Beck et al. (38) was also rated as moderate risk for failing to assess whether fractures had fully healed based on imaging reports; all other studies were classified as low risk. Additionally, all studies, except for S. Y. Elsebahy et al. (44) and M. E. Moncada et al. (59), did not show any potential risk of selective reporting bias.

### 3.3 Standard meta-analysis

#### 3.3.1 Pain relief difference

**FIGURE 3:**
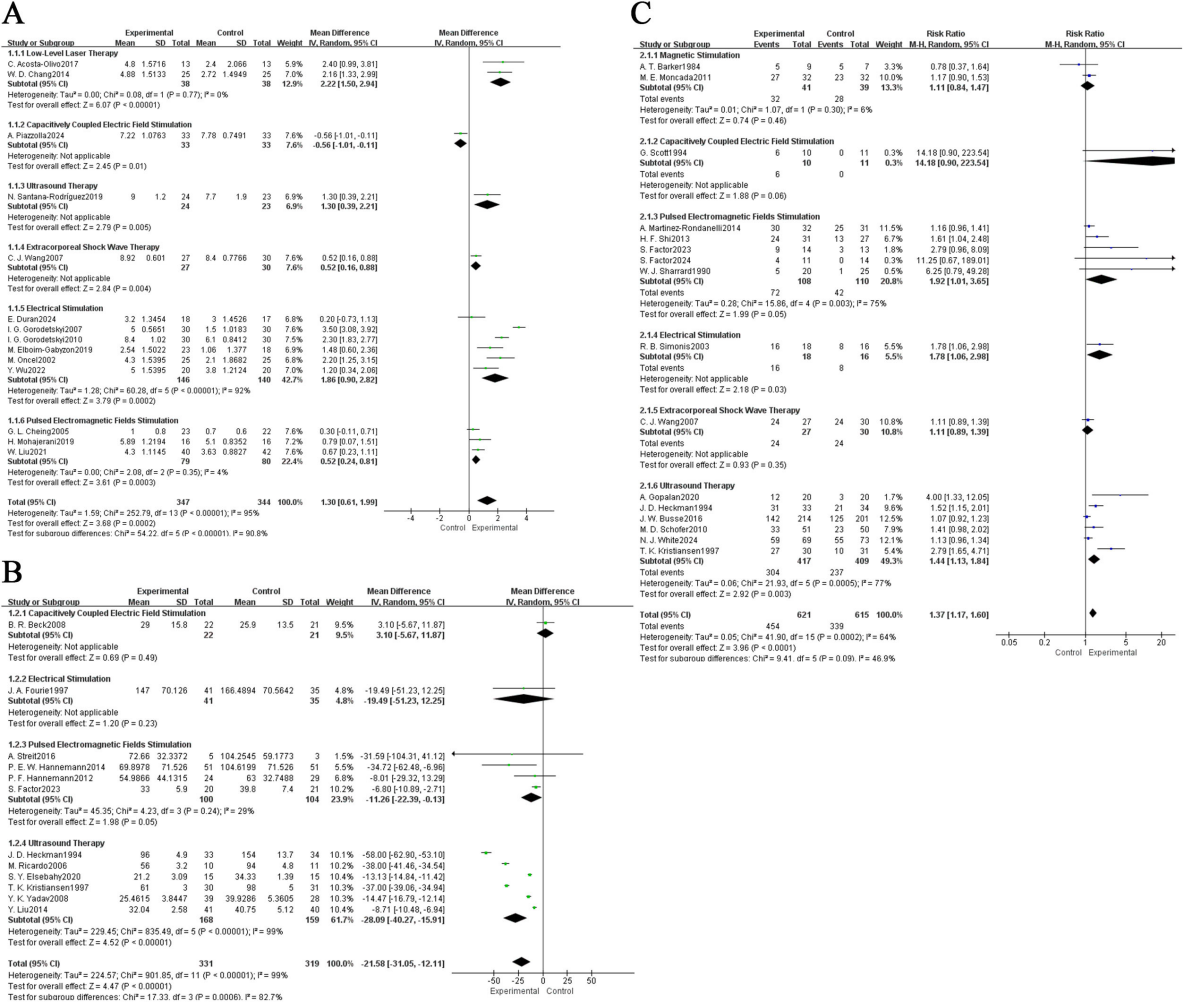
Forest plot of comparison: physical agent modalities group versus control group. **(A)** Difference between post-treatment and pre-treatment pain scores; **(B)** time to complete fracture healing (days); **(C)** number of patients with fully healed fractures.CI, confidence interval; MD, mean difference.

This study included 14 investigations that assessed pain intensity using the Visual Analogue Scale (VAS). To illustrate the extent of pain relief, we used the difference between the VAS score recorded before the application of physical agent modalities and the score obtained after the final treatment session as the primary analytical measure. Due to significant overall heterogeneity (I^2^ = 90.8%, *P* < 0.00001), a random-effects model was employed for the meta-analysis of pain scores. The analyses were further divided into six subgroups based on different physical interventions (LLLT, CCEFS, UST, ESWT, ES, PEMFS) (Figure 3A). For ES, I^2^ was found to be 92% (*P* < 0.00001), while LLLT and PEMFS reported I^2^ values of 0% and 4%, respectively. Due to the limited number of studies, I^2^ could not be calculated for the remaining three interventions. Hence, the variation in different physical agent modalities may have contributed to the high heterogeneity observed. The analysis results demonstrated that LLLT significantly reduced pain in fracture patients: MD = 2.22, 95% CI (1.50, 2.94), *P* < 0.00001. This was followed by ES: MD = 1.86, 95% CI (0.90, 2.82), *P* = 0.0002; UST: MD = 1.30, 95% CI (0.39, 2.21), *P* = 0.005; PEMFS: MD = 0.52, 95% CI (0.24, 0.81), *P* = 0.0003; ESWT: MD = 0.52, 95% CI (0.16, 0.88), *P* = 0.004. Lastly, CCEFS showed an MD of −0.56, 95% CI (−1.01, −1.11), *P* = 0.01, indicating that CCEFS did not alleviate pain, this may be largely attributed to two factors: First, the number of studies included in the analysis was limited, with only one investigation evaluating the analgesic effect of CCEFS, resulting in insufficient statistical power. Second, that study adopted a follow-up period of up to 6 months; although pain levels showed marked improvement over time, the dominant role of the body’s own repair mechanisms during the natural fracture healing process may have substantially diluted the additional effects of physical agent therapy in the later stages. This could be a key reason for the negative effect observed. Future high-quality studies with shorter follow-up periods, particularly during the acute phase, are recommended to clarify the true effectiveness of CCEFS in pain management. In the sensitivity analysis, excluding individual studies did not lead to significant changes in the overall results, suggesting that the findings are robust (Supplementary Figure 1A).

#### 3.3.2 Time to complete fracture healing (days)

In total, 9 studies were included, reporting on the fracture healing times of patients with different fracture locations following physical agent modalities. Due to the high overall heterogeneity of the included studies (I^2^ = 82.7%, *P* = 0.0006), a random-effects model was employed for the meta-analysis. Furthermore, subgroup analyses were conducted for different physical agent modalities (CCEFS, ES, PEMFS, UST) (Figure 3B). The I^2^ values for PEMFS and UST were 29% and 99%, respectively, while I^2^ could not be calculated for CCEFS and ES due to an insufficient number of studies. Thus, the high heterogeneity may be attributed to the different physical intervention methods employed. The results indicated that the treatment group showed a significant improvement in fracture healing time compared to the control group, UST: MD = −28.09, 95% CI (−40.27, −15.91), *P* < 0.00001; PEMFS: MD = −11.26, 95% CI (−22.39, −0.13), *P* = 0.05; CCEFS: MD = 3.10, 95% CI (−5.67, 11.87), *P* = 0.49; ES: MD = −19.49, 95% CI (−51.23, 12.25), *P* = 0.23. Although CCEFS and ES did not show statistical significance, the overall results indicated that physical agent modalities effectively reduced fracture healing time: MD = −21.58, 95% CI (−31.05, −12.11), *P* < 0.00001. In the sensitivity analysis, the summary results remained stable after the exclusion of individual studies, suggesting that the findings are robust (Supplementary Figure 1B).

#### 3.3.3 Number of cases achieving complete fracture healing

16 included studies reported on the number of cases achieving complete fracture healing. Due to moderately high overall heterogeneity (I^2^ = 46.9%, *P* = 0.09), a random-effects model was still employed. The results indicated that, overall, physical agent modalities significantly increased the number of cases achieving complete fracture healing compared to the control group, with a risk ratio (RR) of 1.37, 95% confidence interval (CI) (1.17, 1.60), *P* < 0.0001. Further subgroup analyses were conducted based on different physical agent modalities (MS, CCEFS, PEMFS, ES, ESWT, UST) (Figure 3C), revealing that PEMFS and UST exhibited statistically significant effects and played a positive role in promoting fracture healing, PEMFS: RR = 1.92, 95% CI (1.01, 3.65), *P* = 0.05; UST: RR = 1.44, 95% CI (1.13, 1.84), *P* = 0.003. In addition, the I^2^ values for MS, PEMFS, and UST were 6%, 75%, and 77%, respectively, indicating that the high heterogeneity may still be attributed to the different physical intervention methods. In the sensitivity analysis, after excluding individual studies, the summary results did not show significant changes, suggesting that the findings are robust (Supplementary Figure 1C).

### 3.4 Network meta-analysis

#### 3.4.1 Network map

**FIGURE 4:**
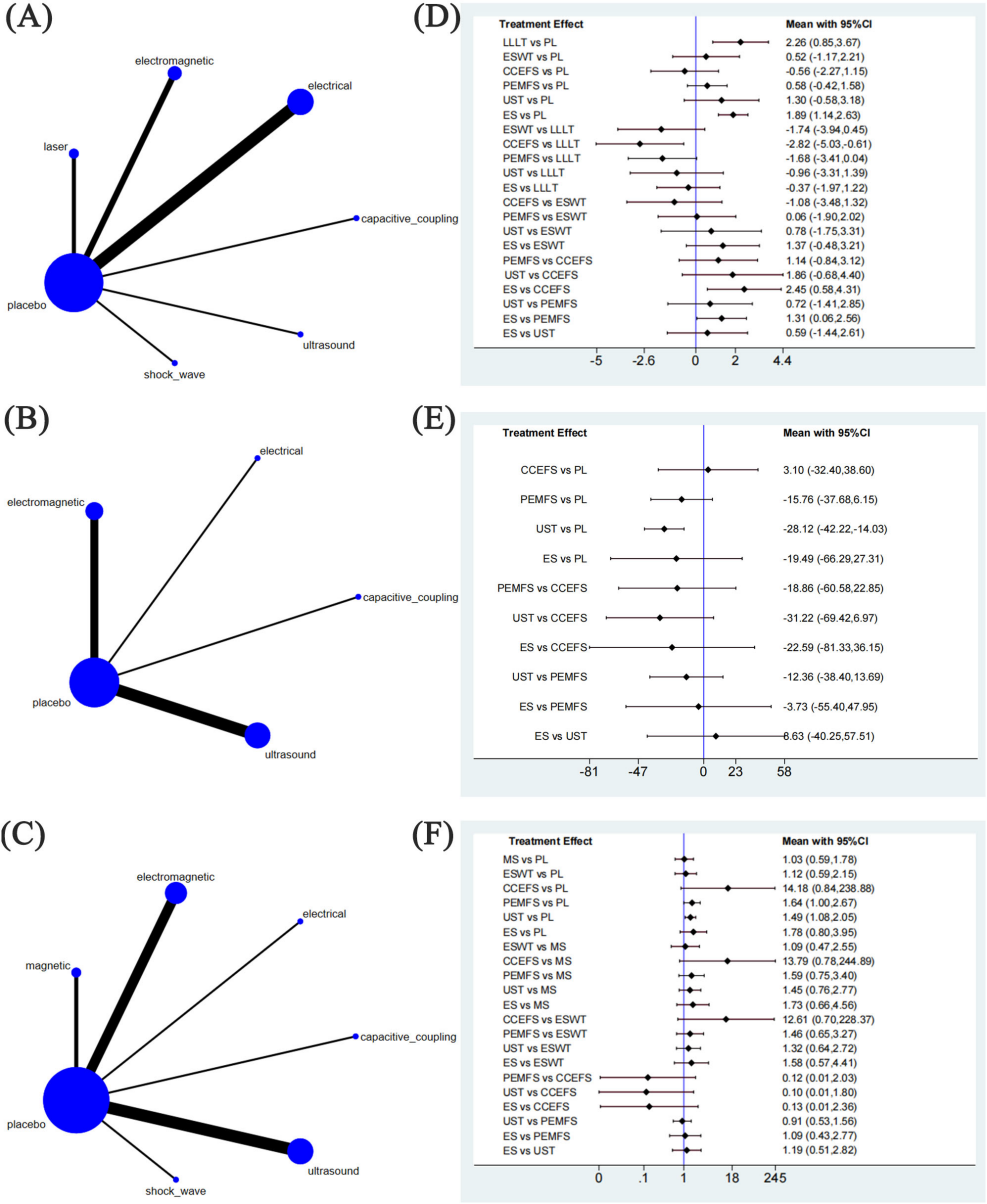
Network meta-analysis of physical agent modalities for fractures. **(A–C)** Network evidence plots for fractures. **(D–F)** Forest plot represents the direct and indirect comparison. PL, placebo; LLLT, low-level laser therapy; MS, magnetic stimulation; ESWT, extracorporeal shock wave therapy; CCEFS, capacitively coupled electric field stimulation; PEMFS, pulsed electromagnetic fields stimulation; UST, ultrasound therapy; ES, electrical stimulation.

We generated three network node diagrams (Figures 4A–C), three primary outcomes, each involving different physical agent modalities. Analysing these study data, we assessed the relative efficacy of seven types of physical agent modalities (Figures 4D–F). Further details are provided in the ranking table (Supplementary Table 2). We conducted pairwise comparative analyses of all treatment approaches using MD and RR with 95% confidence intervals. The results showed that, for most comparisons between physical agent modalities, the 95% confidence intervals included the null value (MD = 0 or RR = 1), indicating that the differences in effectiveness between these interventions did not reach statistical significance. This suggests that the relative efficacy of different physical agent modalities remains uncertain. However, a trend suggesting that Low-Level Laser Therapy (LLLT) may be more effective in alleviating post-fracture pain compared with the other five groups. The following data supports this assertion: ESWT vs LLLT: MD = −1.74, 95% CI (−3.94, 0.45); CCEFS vs LLLT: MD = −2.82, 95% CI (−5.03, –0.61); PEMFS vs LLLT: MD = −1.68, 95% CI (−3.41, 0.04); UST vs LLLT: MD = −0.96, 95% CI (−3.31, 1.39), and ES vs LLLT: MD = −0.37, 95% CI (−1.97, 1.22) (Figure 4D). Similarly, when considering the time to complete fracture healing (days), although no statistically significant differences were observed, UST showed a notable trend toward reducing the time to complete fracture healing (days) compared to the other groups. This trend is supported by the data: UST vs CCEFS: MD = −31.22, 95% CI (−69.42, 6.97); UST vs PEMFS: MD = −12.36, 95% CI (−38.40, 13.69); ES vs UST: MD = 8.63, 95% CI (−40.25, 57.51) (Figure 4E). Moreover, when considering the number of patients achieving complete fracture healing, CCEFS demonstrated a significant advantage over the other intervention groups, CCEFS vs MS: RR = 13.79, 95% CI(0.78, 244.89); CCEFS vs ESWT: RR = 12.61, 95% CI (0.70, 228.37); PEMFS vs CCEFS: RR = 0.12, 95% CI (0.01, 2.03); UST vs CCEFS: RR = 0.10, 95% CI (0.01, 1.80); ES vs CCEFS: RR = 0.13, 95% CI (0.01, 2.36) (Figure 4F).

#### 3.4.2 Ranking of treatments

**FIGURE 5:**
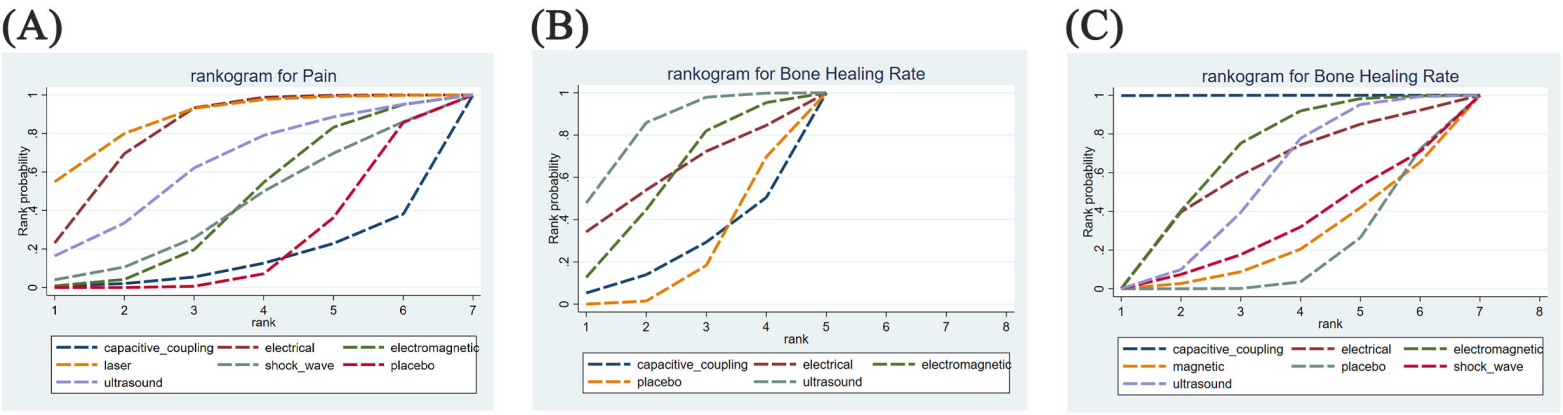
Rankogram for all outcomes. Each line segment represents a treatment. The area enclosed by the line segment and the coordinate axis represents the cumulative probability of treatment. **(A)** Difference between post-treatment and pre-treatment pain scores; **(B)** time to complete fracture healing (days); **(C)** number of patients with fully healed fractures.

Figure 5 illustrates the cumulative probabilities of each intervention across various potential rankings, represented by SUCRA values, which indicate the ranking of treatments; a higher SUCRA value signifies a more favorable ranking among all available treatments. A SUCRA value of 100% denotes the best treatment effect, while a SUCRA value of 0% indicates the poorest treatment effect. According to the ranking results shown in Table 2, the two highest-ranked interventions for pain relief are LLLT (SUCRA 87.5%) and ES (SUCRA 80.8%), followed by UST (SUCRA 62.5%), PEMFS (SUCRA 42.9%), ESWT (SUCRA 41.0%), and CCEFS (SUCRA 13.7%). In terms of time to complete fracture healing (days), UST (SUCRA 82.9%) and ES (SUCRA 61.3%) are ranked highest, followed by PEMFS (SUCRA 58.7%) and CCEFS (SUCRA 24.7%). Finally, with respect to the number of patients achieving complete fracture healing, CCEFS (SUCRA 99.9%) and PEMFS (SUCRA 67.6%) ranked highest, followed by ES (SUCRA 58.4%), UST (SUCRA 53.6%), ESWT (SUCRA 30.2%), and MS (SUCRA 23.2%).

**TABLE 2:** SUCRA ranking of different outcome indicators.

Physical agent modalities	Pain relief difference	Time to complete fracture healing (days)	Number of cases achieving complete fracture healing
Capacitive_coupling	13.7%	24.7%	99.90%
Electrical	80.8%	61.3%	58.40%
Electromagnetic	42.9%	58.7%	67.60%
Laser	87.5%	−	−
Placebo	21.7%	22.4%	17.10%
Shock_wave	41.0%	−	30.20%
Ultrasound	62.5%	82.9%	53.60%
magnetic	−	−	23.20%

### 3.5 Publication of bias

**FIGURE 6:**
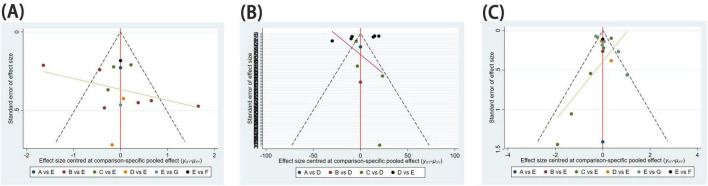
Funnel diagram. Publication bias for assessing study results. **(A)** Difference between post-treatment and pre-treatment pain scores; **(B)** time to complete fracture healing (days); **(C)** number of patients with fully healed fractures.

Funnel plots were employed to assess publication bias for all outcome indicators. The funnel plots for Pain Relief Difference, Time to Complete Fracture Healing (days), and Number of Cases Achieving Complete Fracture Healing exhibited a symmetrical and even distribution, suggesting the absence of significant publication bias (Figure 6).

## 4 Discussion

To the best of our knowledge, this study represents the first NMA comparing the efficacy of different physical agent modalities for treating patients with fractures. This NMA meticulously reviewed the most recent data from 39 eligible randomized controlled trials, evaluating the effectiveness of physical agent modalities in fracture patients. This study confirms that physical agent modalities significantly promotes fracture healing and alleviates pain. To compare the effects of different physical agent modalities, we sought indirect evidence through pairwise comparisons. However, indirect treatment comparisons revealed no statistically significant differences in efficacy among the seven modalities. Subsequently, by calculating the SUCRA values for these therapies and conducting a ranking analysis, we found that both LLLT and ES significantly relieve pain, with the reduction in pain scores likely reaching or even exceeding the minimal clinically important difference. This indicates that their effects go beyond statistical significance and provide pain relief that is genuinely meaningful to patients. Such a degree of improvement can help enhance sleep quality and daily functioning, thereby improving overall quality of life and potentially reducing dependence on analgesic medications. Consequently, these two physical agent modalities demonstrate substantial clinical value as complementary approaches to pain management. UST markedly shortens fracture healing time, while CCEFS significantly increases the healing rate in patients with fractures, followed by PEMFS. These effects enable patients to regain physical function earlier, fundamentally reducing the risk of complications associated with prolonged immobilization or delayed healing, and ultimately leading to substantial savings in healthcare resources. In this context, these physical agent modalities serve not only as effective clinical tools for preventing fracture non-union but also as strategies that enhance the overall quality of treatment. They therefore provide strong evidence supporting the clinical prioritization of efficient physical therapy modalities. However, since only a few studies have examined the application of LLLT and CCEFS in patients with fractures, this result warrants cautious interpretation.

Patients with fractures often experience acute postoperative pain, which is typically managed with analgesic medications. However, such treatments frequently come with adverse side effects, including nausea, vomiting, delirium, constipation, and gastrointestinal dysfunction. The incidence of these side effects is particularly higher in the elderly population (75), and postoperative analgesic efficacy is often poorer compared to younger individuals (76). Consequently, the application of non-pharmacological and non-invasive analgesic methods in the management of acute postoperative pain has garnered increasing attention. Our research found that low-level laser therapy (LLLT) exhibits the best analgesic effects. However, due to the low certainty of evidence, further studies are required to validate this conclusion. LLLT is based on a specialized technical device capable of emitting light beams with precise characteristics for medical applications. It stimulates the mitochondria to produce ATP, enhances mitochondrial electron transport rates, regulates reactive oxygen species to reduce oxidative stress, and induces the activation of transcription factors such as AP-1, p53, NF-kB, and HIF, thereby promoting extracellular matrix deposition and activating anti-inflammatory and anti-apoptotic pathways. Clinically, these effects manifest as pain and inflammation relief, as well as facilitation of tissue repair (77–79). Literature indicates that *in vitro*, LLLT modulates the inflammatory response by activating the WNT pathway and inhibiting the NF-kB signaling pathway (80). *In vivo*, it regulates the levels of inflammatory precursor factors such as IL-1, IL-6, IL-8, and IL-18 to control the inflammatory response (81). Furthermore, LLLT positively influences bone tissue metabolism and fracture healing (82, 83) by stimulating microcirculation and increasing the activity of osteoblasts, thus enhancing the osteogenic effect (84). However, therapeutic benefits appear restricted to early healing phases (< 21 days post-fracture), with diminished efficacy in chronic non-union models.(85).

Beyond LLLT, electrical stimulation (ES) encompasses established analgesic approaches such as transcutaneous electrical nerve stimulation (TENS), non-invasive interactive neurostimulation (NIN), and interferential current (IFC). TENS delivers pulsed electrical currents transcutaneously, making it a prevalent non-pharmacological intervention for pain management (86, 87). It is capable of generating a sensation similar to acupuncture at frequencies of 2–4 Hz by stimulating Aδ and C fibers in the afferent nerves, thereby activating downstream pain inhibition pathways and producing a spatially diffuse analgesic effect (88). Studies by Gorodetskyi I et al. (49) and Lord SR et al. (89) have investigated the role of TENS in reducing acute postoperative pain in elderly patients following hip fractures, both reporting significant pain relief, which is consistent with our findings. Moreover, NIN has been shown to have a positive effect in the postoperative care of patients with femoral neck fractures (49), with its pain relief mechanism thought to involve segmental and descending neural inhibition (90). IFC operates on the principle of a low-frequency-modulated medium-frequency current created by the superposition of two medium-frequency currents with slight phase differences (91). IFC therapy is believed to alleviate pain through gate control mechanisms and the release of endogenous opioids (92). Although this therapy has been in use for decades, its physiological effects have not been fully substantiated, making it challenging to completely elucidate its analgesic action (93).

Fracture patients frequently endure acute postoperative pain, conventionally managed with analgesics. However, these pharmacological interventions often induce adverse effects such as nausea, constipation, delirium, and gastrointestinal dysfunction. According to our study, physical agent modalities significantly shortens the time to fracture healing and improves the complete healing rate in fracture patients. SUCRA ranking in this study indicates that ultrasound therapy (UST) can markedly reduce the time to complete fracture healing (days) compared to other physical agent modalities, which aligns with the findings of Kristiansen et al. (54) who reported that treatment with low-intensity pulsed ultrasound (LIPUS) shortened radiological healing times by 38%. When ultrasound propagates through biological tissues, it generates micro-mechanical strain, which stimulates biochemical responses at the cellular level and promotes bone formation (94). Fracture patients often experience prolonged immobilization, leading to a deficiency of mechanical load at the injury site. However, ultrasound can produce mechanical forces that improve the mechanical environment of the affected area, potentially facilitating endochondral ossification, a key mechanism in fracture healing (95, 96). Additionally, the mechanical stress generated by ultrasound further promotes osteogenesis, protein synthesis, calcium uptake, and DNA synthesis in various cell types (97). As ultrasound transmits through the tissue to the bone, cells adjacent to the fracture site convert biomechanical stimuli into biochemical responses via integrins, which serve as crucial molecular mediators of mechanotransduction (98). Furthermore, ultrasound stimulation increases the expression of integrins, enhancing the adhesion of osteoblasts at the fracture site, thereby aiding in fracture healing (99, 100).

Early clinical studies suggest that high-intensity ultrasound stimulation in the range of 5000 to 25000 mW/cm^2^ may induce adverse effects including necrosis, cessation of bone healing, and fibrous tissue formation (101, 102). Consequently, low-intensity pulsed ultrasound (LIPUS) has consequently become the clinical standard (103). LIPUS has been shown to positively influence fracture healing regardless of the patient’s age, smoking status, the presence of a fracture gap, fibular fractures, or the location of distal fractures (104). Animal studies indicate that LIPUS not only accelerates the formation of bone callus but also enhances the mechanical strength at the fracture site (105, 106). During the fracture healing process, The periosteum serves as a primary reservoir for osteoprogenitor cells during bone regeneration, playing a central role in callus formation. Tam et al. (107) demonstrated that LIPUS interventions positively stimulate osteogenesis and the activation of cell differentiation in human periosteal cells. Additionally, cyclooxygenase-2 (COX-2) and prostaglandin E2 (PGE2) are key biological processes involved in the mineralization and remodeling phases of bone healing (108), COX-2 promotes fracture healing by upregulating genes associated with endochondral ossification and angiogenesis (98, 109), whereas PGE2 enhances collagen synthesis in cultured bone and further stimulates osteoblast proliferation (110). Tang et al. (99) and Kokubu T et al. (111) found that expression of COX-2 and PGE2 in osteoblasts was significantly increased when cells were subjected to ultrasound stimulation.

Pulsed Electromagnetic Fields Stimulation (PEMFS) has emerged as a clinical mainstay (2), demonstrating particular efficacy during the angiogenic-osteogenic coupling phase of bone repair and remodeling (112). Multiple studies have demonstrated that PEMFS actively promotes bone healing by regulating voltage-gated ion channels, increasing cytosolic calcium ion concentrations, enhancing early angiogenesis, and facilitating the differentiation and maturation of osteoblasts (113). Research indicates that PEMFS upregulates TGF-β expression and promotes the proliferation and osteogenic differentiation of stem cells via coordinated signaling through BMP, ERK/MAPK, and Notch pathways (114–117). The effectiveness of PEMFS is closely related to exposure duration, to significantly enhance fracture healing, PEMFS should be applied for at least 8 h per day over a period of 45–60 days (118). Furthermore, studies have found that PEMFS can upregulate the expression of placental growth factor (PIGF) and brain-derived neurotrophic factor (BDNF). PIGF, a member of the vascular endothelial growth factor (VEGF) subfamily, is a key regulator of angiogenesis and vasculogenesis (119). BDNF promotes angiogenesis through two mechanisms: Firstly, by locally activating subsets of endothelial cells, and secondly, by recruiting bone marrow-derived cells. These both mechanisms contribute to the formation of new blood vessels, thereby facilitating bone formation (120). Additionally, Parhampour et al. (121) discovered that PEMFS can improve bone metabolic disorders and restore joint function.

In our study, CCEFS demonstrated significant effects on fracture healing and was ranked first in the third outcome measure based on the SUCRA rankings. As a non-invasive bone growth stimulation method, CCEFS has the potential to enhance osteoblast function and increase new bone formation (122). An *in vitro* study revealed the mechanism of action of CCEFS, which involves the activation of voltage-gated calcium channels in the plasma membrane, leading to increased cytosolic calcium concentration and phospholipase A2 (PLA2) activity (123). The rise in cytosolic calcium activates the calmodulin pathway, further upregulating the expression of osteogenic-related genes, including fibroblast growth factor (FGF) 2, osteocalcin (OCN), TGF-β, BMP, and alkaline phosphatase (ALP) (124, 125). PLA2 promotes the synthesis of PGE2, thereby further facilitating the osteogenic process (126). Additionally, a study (127) have reported that CCEFS has a positive impact on alleviating chronic pain. However, our NMA did not demonstrate a significant effect of CCEFS on post-fracture pain relief. This may be attributed to the inclusion of only one relevant study (62), which, although indicating that CCEFS could more rapidly relieve pain, found no significant difference in the overall level of pain relief compared to the control group.

In the included studies, the number of investigations on LLLT, MS, ESWT, and CCEFS was relatively limited, largely reflecting the characteristics of each technique and their current clinical use. ESWT carries a potential risk of secondary injury due to possible adverse effects such as hematoma formation and increased pain (128). Research on MS has focused mainly on neurological rehabilitation (129), with comparatively less application in fracture treatment. CCEFS already has a well-established therapeutic protocol for fracture healing (122), and its technical stability has resulted in fewer novel research directions. For LLLT, progress in fracture pain management has been slow, partly due to the lack of standardization in pain assessment tools (such as VAS, NRS, and the McGill Pain Questionnaire) and partly because current clinical practice still relies heavily on pharmacological analgesia (130). These factors may together contribute to the current relative scarcity of research on these therapies in the field of fracture rehabilitation.

## 5 Limitation

First, restricted study availability and underpowered sample sizes compromised generalizability while reducing statistical precision. Second, there was an imbalance in the number of comparisons and sample sizes among the physical agent modalities. Among the three different outcome measures, studies on UST constituted the largest proportion, while those on CCEFS and MS comprised the smallest, which may have impacted the research findings. Third, we acknowledge the substantial heterogeneity observed in this study, which may largely stem from clinical differences among the included trials, such as variations in fracture type, duration of intervention, and device parameters. Because the original studies provided insufficient data, we were unable to perform subgroup analyses to further explore the specific influence of these factors. Therefore, the findings of this network meta-analysis should be interpreted as representing an overall effect across diverse clinical contexts. Future studies should adopt more standardized designs and provide more detailed reporting to better account for these key variables. Fourth, the lack of direct comparisons between physical agent modalities, relying instead on indirect evidence, may limit the reliability and comprehensiveness of the conclusions. Finally, the use of SUCRA scores does not account for differences in study quality and relies solely on relative ranking to evaluate treatment efficacy. Including low-quality studies may introduce bias and lead to systematic errors in effect size estimation. When such biased estimates are incorporated into a network meta-analysis model, they can distort the true comparative effectiveness between treatments, causing the SUCRA rankings to deviate from reality and reducing their overall reliability. Given that the design of the SUCRA scoring system focuses on relative efficacy while neglecting effect size, it may inadvertently undermine the clinical significance of the treatment effects.

## 6 Conclusion

Physical agent modalities demonstrate therapeutic potential in fracture management, effectively reducing pain and enhancing osseous regeneration. In indirect head-to-head comparisons, although different physical agent modalities did not show clear advantages or disadvantages in pain relief and fracture healing, this study provides valuable insights for clinical decision-making. Notably, LLLT and ES displayed potential advantages in pain alleviation, while UST and CCEFS exhibited superior effectiveness in promoting fracture healing. However, these preliminary conclusions require validation through high-quality, large-sample randomized controlled trials, and further clinical research is necessary to confirm the efficacy of these interventions.

## Data Availability

The original contributions presented in this study are included in this article/Supplementary material, further inquiries can be directed to the corresponding authors.

## Footnotes

1
http://www.njbinbin.net/topics/ZH_CN/392/references/

2
https://methods.cochrane.org/risk-bias-2
