# Supplementary material for: Evaluation of the efficacy of physical agent modalities in patients with fractures: a systematic review and network meta-analysis
Source: Front Med (Lausanne). 2025 Oct 29;12:1646903. doi: 10.3389/fmed.2025.1646903 (PMC12614468; doi:10.3389/fmed.2025.1646903)
Supplement: Supplementary file 1 [file Table_1.docx]

**Supplementary Table 1.** **Detailed search strategy**

| **Pubmed (Medline)** | |
| --- | --- |
| **#1** | electrical stimulation [All Fields] OR electromagnetic[All Fields] OR ultrasound therapy[All Fields] OR laser[All Fields] OR magnetic[All Fields] OR shock wave[All Fields] OR **capacitive coupling**[All Fields] |
| **#2** | pain[All Fields] OR **bone regeneration**[All Fields] **OR regeneration of bone**[All Fields] **OR bone defect regeneration**[All Fields] **OR bone formation**[All Fields] **OR osteogenesis**[All Fields] **OR osteogenic**[All Fields] **OR bone reconstruction**[All Fields] **OR bone repair**[All Fields] **OR bone healing**[All Fields] |
| **#3** | fracture[All Fields] |
| **#1 AND #2 AND #3,Document type=Clinical Trial OR Randomized Controlled Trial** | |
| **Embase** | |
| **#1** | ‘electrical stimulation’/exp OR ‘electromagnetic’/exp OR ‘ultrasound therapy’/exp OR ‘laser’/exp OR ‘magnetic’/exp OR ‘shock wave’/exp OR ‘**capacitive coupling**’/exp |
| **#2** | ‘pain’/exp OR ‘**bone regeneration**’/exp **OR** ‘**regeneration of bone**’/exp **OR** ‘**bone defect regeneration**’/exp **OR** ‘**bone formation**’/exp **OR** ‘**osteogenesis**’/exp **OR** ‘**osteogenic**’/exp **OR** ‘**bone reconstruction**’/exp **OR** ‘**bone repair**’/exp **OR** ‘**bone healing**’/exp |
| **#3** | ‘fracture’/exp |
| **#1 AND #2 AND #3,Document type=Clinical Trial OR Randomized Controlled Trial** | |
| **Web of Science** | |
| **#1** | TS= (electrical stimulation OR electromagnetic OR ultrasound therapy OR laser OR magnetic OR shock wave OR **capacitive coupling**) AND fracture AND (pain OR **bone regeneration OR regeneration of bone OR bone defect regeneration OR bone formation OR osteogenesis OR osteogenic OR bone reconstruction OR bone repair OR bone healing)** |
| **#2** | TI=(electrical stimulation OR electromagnetic OR ultrasound therapy OR laser OR magnetic OR shock wave OR **capacitive coupling**) AND fracture AND (pain OR **bone regeneration OR regeneration of bone OR bone defect regeneration OR bone formation OR osteogenesis OR osteogenic OR bone reconstruction OR bone repair OR bone healing)** |
| **#3** | AB=(electrical stimulation OR electromagnetic OR ultrasound therapy OR laser OR magnetic OR shock wave OR **capacitive coupling**) AND fracture AND (pain OR **bone regeneration OR regeneration of bone OR bone defect regeneration OR bone formation OR osteogenesis OR osteogenic OR bone reconstruction OR bone repair OR bone healing)** |
| **#1 AND #2 AND #3,Document type=Clinical Trial** | |
