# Supplementary material for: Evaluation of the efficacy of physical agent modalities in patients with fractures: a systematic review and network meta-analysis
Source: Front Med (Lausanne). 2025 Oct 29;12:1646903. doi: 10.3389/fmed.2025.1646903 (PMC12614468; doi:10.3389/fmed.2025.1646903)
Supplement: Supplementary file 2 [file Table_2.docx]

Supplementary Table 2A. pain relief difference Network Meta-Analysis Results

| capacitive_coupling |  |  |  |  |  |  |
| --- | --- | --- | --- | --- | --- | --- |
| -2.45 (-4.83, 0) | electrical |  |  |  |  |  |
| -1.14 (-3.71, 1.44) | 1.32 (-0.34, 2.87) | electromagnetic |  |  |  |  |
| -2.82 (-5.64, 0) | -0.37 (-2.37, 1.55) | -1.68 (-3.83, 0.47) | laser |  |  |  |
| -0.56 (-2.79, 1.67) | 1.89 (0.91, 2.8) | 0.58 (-0.72, 1.88) | 2.26 (0.55, 3.99) | placebo |  |  |
| -1.08 (-4.22, 2.07) | 1.37 (-1.06, 3.75) | 0.06 (-2.5, 2.67) | 1.74 (-1.04, 4.56) | -0.52 (-2.74, 1.71) | shock_wave |  |
| -1.86 (-5.1, 1.38) | 0.59 (-1.97, 3.09) | -0.73 (-3.42, 1.98) | 0.96 (-1.94, 3.87) | -1.3 (-3.66, 1.06) | -0.79 (-4.03, 2.44) | ultrasound |

Supplementary Table 2B. Time to complete fracture healing Network Meta-Analysis Results

| capacitive_coupling |  |  |  |  |
| --- | --- | --- | --- | --- |
| 22.58 (-49.25, 94.48) | electrical |  |  |  |
| 18.85 (-33.06, 73.51) | -3.6 (-64, 58.81) | electromagnetic |  |  |
| 3.07 (-43.12, 49.14) | -19.58 (-74.76, 35.74) | -15.95 (-43.66, 9.8) | placebo |  |
| 31.08 (-18.25, 80.7) | 8.5 (-49.64, 66.81) | 12.19 (-21.46, 43.67) | 28.1 (9.46, 46.86) | ultrasound |

Supplementary Table 2C. Number of fully healed fractures Network Meta-Analysis Results

| capacitive_coupling |  |  |  |  |  |  |
| --- | --- | --- | --- | --- | --- | --- |
| 1.013e+11 (14.81, 1.181e+36) | electrical |  |  |  |  |  |
| 9.096e+10 (13.6, 1.06e+36) | 0.94 (0.2, 3.27) | electromagnetic |  |  |  |  |
| 1.774e+11 (26.42, 2.227e+36) | 1.78 (0.42, 8.13) | 1.88 (0.76, 6.85) | magnetic |  |  |  |
| 1.791e+11 (27.74, 2.259e+36) | 1.83 (0.56, 6.24) | 1.94 (1.14, 4.51) | 1.03 (0.43, 2.38) | placebo |  |  |
| 1.606e+11 (23.67, 1.887e+36) | 1.63 (0.32, 8.49) | 1.71 (0.56, 7.45) | 0.91 (0.22, 3.61) | 0.89 (0.29, 2.69) | shock_wave |  |
| 1.15e+11 (17.34, 1.362e+36) | 1.17 (0.31, 4.16) | 1.24 (0.62, 3.15) | 0.66 (0.23, 1.64) | 0.64 (0.37, 0.98) | 0.73 (0.2, 2.28) | ultrasound |
