# Supplementary figures and images for: Evaluation of the efficacy of physical agent modalities in patients with fractures: a systematic review and network meta-analysis
Source: Front Med (Lausanne). 2025 Oct 29;12:1646903. doi: 10.3389/fmed.2025.1646903 (PMC12614468; doi:10.3389/fmed.2025.1646903)

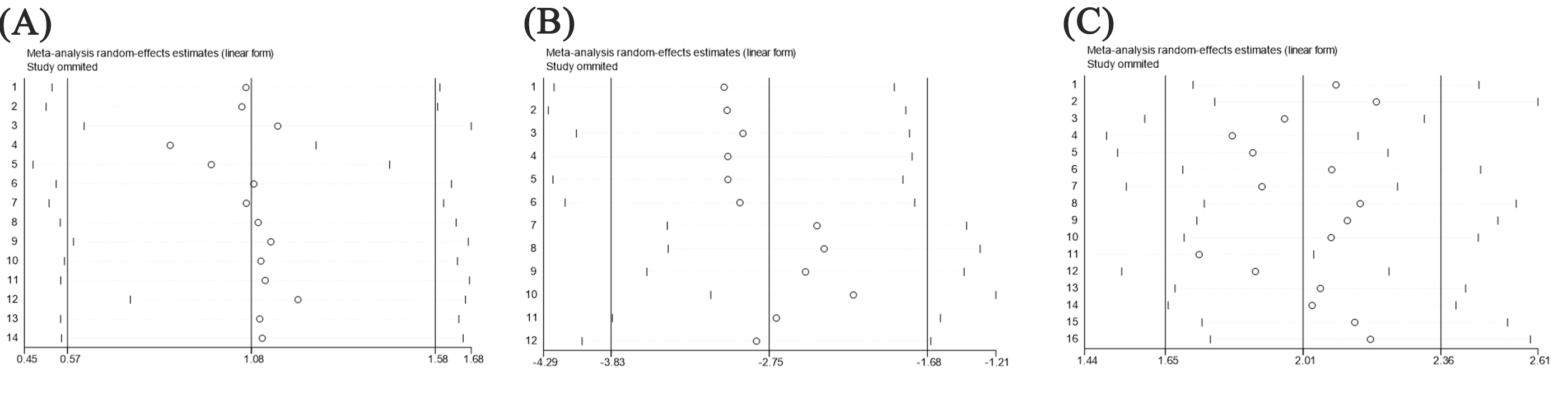

Supplement: Supplementary file 3 [file Image_1.tif]
